# Efficacy and factors affecting the choice of enucleation and liver resection for giant hemangioma: a retrospective propensity score-matched study

**DOI:** 10.1186/s12893-020-00935-0

**Published:** 2020-11-07

**Authors:** Mingguang Ju, Feng Xu, Wenyan Zhao, Chaoliu Dai

**Affiliations:** Department of General Surgery, Shengjing Hospital of China Medical University, No.36 Sanhao Street, Heping District, Shenyang, 110004 Liaoning China

**Keywords:** Hepatic hemangioma, Enucleation, Liver resection, Propensity score matching, Surgery

## Abstract

**Background:**

Liver resection (LR) and enucleation (EN) are the main surgical treatment for giant hepatic hemangioma (HH), but how to choose the type of surgery is still controversial. This study aimed to explore the efficacy and the factors affecting the choice of open procedure for HH.

**Methods:**

The data for patients with pathologically confirmed HH who underwent open surgery from April 2014 to August 2020 were analyzed retrospectively. Univariate and multivariate analyses with logistic regression were performed to disclose the factors associated with the choice of EN or LR. Propensity score matching (PSM) analysis was used to compare the efficacy of the two procedures.

**Results:**

A total of 163 and 110 patients were enrolled in the EN and LR groups. Following 1:1 matching by PSM analysis, 66 patients were selected from each group. Centrally located lesions (OR: 0.131, 95% CI 0.070–0.244), tumors size > 12.1 cm (OR: 0.226, 95% CI 0.116–0.439) and multiple tumors (OR: 1.860, 95% CI 1.003–3.449) were independent factors affecting the choice of EN. There was no significant difference in the median operation time (156 vs. 195 min, P = 0.156), median blood loss (200 vs. 220 ml, P = 0.423), blood transfusion rate (33.3% vs. 33.3%, P = 1.000), mean postoperative feeding (3.1 vs. 3.3 d, P = 0.460), mean postoperative hospital stay (9.5 vs. 9.0 d, P = 0.206), or the major complication rates between the two groups.

**Conclusions:**

Peripherally located lesions, tumors size ≤ 12.1 cm and multiple tumors were more inclined to receive EN. There was no significant difference in the efficacy of EN or LR.

## Background

Hepatic hemangioma (HH) is the most common benign tumor of the liver [1]. Most patients with HH are asymptomatic and the lesions are incidentally detected on ultrasound, computed tomography (CT) or magnetic resonance imaging (MRI) [2]. Small asymptomatic HH (generally < 5 cm) can be followed up without treatment. However, some patients with giant HH (≥ 5 cm) may develop clinical symptoms or complications such as abdominal pain, jaundice, nausea, vomiting. A small number of patients may develop Kasabach–Merritt syndrome due to platelet destruction in the hemangioma or spontaneous rupture [3–6].

Surgical resection is the main treatment strategy for symptomatic giant HH [7–9]. The surgical treatment for HH includes open, laparoscopic, or robotic liver resection (LR) and enucleation (EN) [2, 10]. EN was first proposed in 1988 [11]. Some surgeons think that there are few blood vessels in the interface between HH and liver parenchyma, and it can be bluntly separated along with the interface so the purpose of less bleeding and complete removal of the focus can be achieved, which means EN is simpler and safer than the traditional LR [12–14], especially for HH at special locations, such as tumors near the hepatic hilum [15]. However, some studies found that there was no significant difference in the curative effect or postoperative outcome between the two techniques [16–18]. The characteristics of tumors in the previous studies were quite different [12–14, 19]. In the studies that supported EN, the mean tumor diameter was about 5 cm, and most of them were smaller than 10 cm [12–14]. A few studies included patients with very large hemangiomas and had contradictory results [19]. Therefore, the choice of the open surgical method for hemangioma cannot be decided based on these studies. Given the controversial results in the past, there are currently no guidelines for the choice between EN and LR. This study was conducted to evaluate the outcomes of the two open procedures and explore the factors affecting the choice of the two methods.

## Methods

### Patient selection

The clinical data of 337 patients with HH confirmed by postoperative pathology in Department of General Surgery at Shengjing Hospital of China Medical University from April 2014 to August 2020 was retrospectively collected. This study was approved by the Ethics Review Committee of Shengjing Hospital, and all patients or their authorizers gave written informed consent before the operation. The inclusion criteria were as follows: (i) patients only received open LR or EN; (ii) patients with Child–Pugh A; (iii) postoperative pathology confirmed to be cavernous hemangioma. The exclusion criteria: (i) patients underwent both EN and LR; (ii) laparoscopic or robotic surgery; (iii) accompanied by serious systemic diseases; (iv) patients received preoperative transarterial embolization or any other non-surgical treatment for HH; (v) patients who underwent EN initially but were later converted to LR.

### Preoperative evaluation

**Fig. 1:**
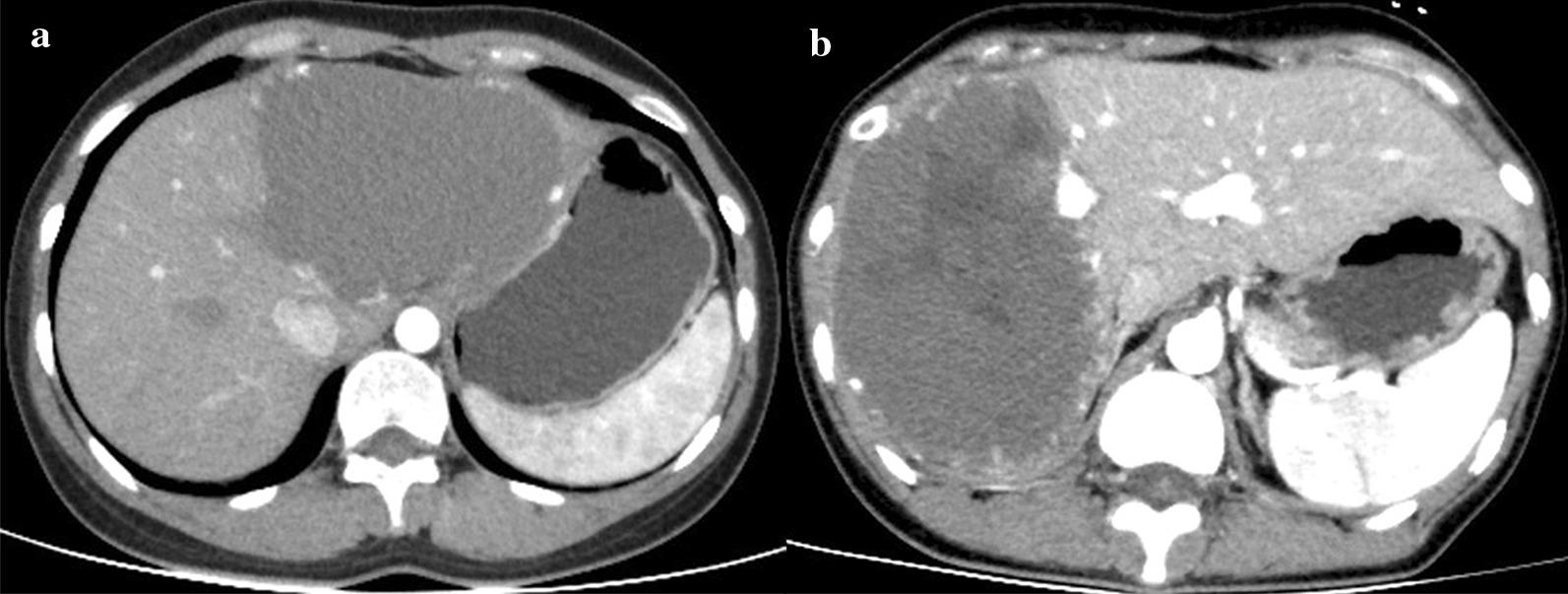
Contrast-enhanced computed tomography of the abdomen: **a** A peripherally located hemangioma (S2 and S3) excised by EN. **b** A centrally located hemangioma (S7 and S8) excised by right hemihepatectomy

Preoperative evaluation included detailed clinical history, physical examination, laboratory tests, radiological investigations including ultrasound, contrast enhanced CT and/or magnetic resonance imaging, and indocyanine green 15-min retention test (ICGR15). For large tumors and those with a close relationship with intrahepatic blood vessels or the inferior vena cava, three-dimensional visualization was applied to evaluate the location of the tumor and its relationship with blood vessels. The peripherally located HHs were defined as HHs located in S2, 3, 4b, 5, and 6. The esions located in S1, 4a, 7, and 8 were defined as the centrally located HHs (Fig. 1). HHs near to or even compressing the trunk or main branches of the portal vein, hepatic vein or hepatic artery were regarded as tumors proximal to the massive vessels. Additional relevant tests such as gastroscopy were performed to exclude other digestive tract-related diseases in some patients.

### Surgical procedures and perioperative management

The patients in both groups received general anesthesia with endotracheal intubation. A right subcostal incision or a right transabdominal rectus incision was used to enter the abdomen and liver mobilization was done according to the location of the tumor.

1. EN—Dissection was performed along with the tumor capsule all around the tumor in the plane between the tumor and liver tissue without the loss of any normal hepatic parenchyma. The blood vessels and bile ducts entering or leaving the tumor were ligated and divided.
2. LR—For non-anatomical liver resection, the line of parenchymal transection was marked about 1–2 cm away from the tumor over the liver capsule using electrocautery. The liver parenchyma was divided with the classic clamp crushing technique. The larger intervening ducts and vessels of the liver were ligated and small diameter vessels were electrocoagulated until the liver tumor was completely removed.

In some cases, intermittent Pringle maneuver was used to reduce bleeding. Anatomical liver resection includes resection of Couinaud’s segments, sectionectomy, and hemihepatectomy. An intraoperative ultrasound would be used to ensure complete resection of the tumor if necessary.

All patients received similar postoperative management. Ultrasound or CT scans were performed one week after the operation. The laboratory tests were examined every other day. The major perioperative complications were recorded and defined using the definition suggested by the International Study Group of Liver Surgery (ISGLS) [20, 21].

### Statistical analysis

**Fig. 2:**
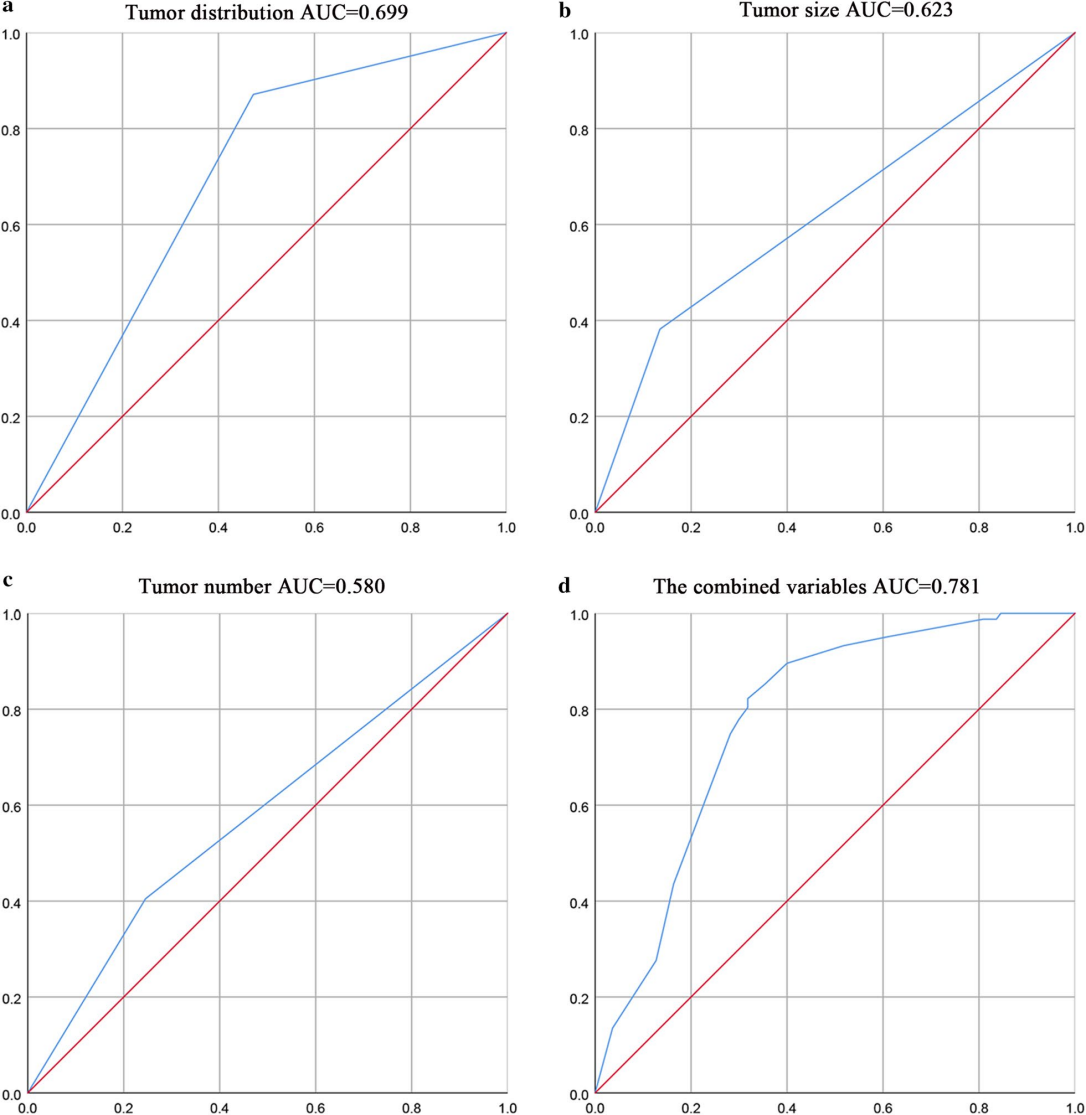
Predictive efficacy of the three variables and the combined variable. The AUCs for the choice of the surgical methods were 0.699, 0.623, 0.580 for the variables: tumor distribution, tumor diameter, and tumor number, respectively (**a**–**c**). Furthermore, the AUC of the combined variables was 0.781 (**d**)

The continuous variables with normal distribution are expressed as mean ± standard deviation (*SD*), and the non-normal continuous variables are expressed as median (interquartile range). The independent sample *t*-test was used to compare the continuous normal distribution variables. The Mann–Whitney *U* test was used to compare the continuous non-normal distribution variables. The Pearson Chi-square test or Fisher exact probability method was used to comparing the categorical data. Baseline variables with a *P* < 0.05 on univariate analysis were included in the multivariate analysis. Logistic regression was performed to determine the independent factors associated with the choice of the surgical methods. Subsequently, the receiver operating characteristic (ROC) curve was graphed with the probabilities, and the area under the curve (AUC) was used to evaluate the efficacy of the multivariate combined model (Fig. 2). The calibration of the model was evaluated by the Hosmer–Lemeshow good of fit test. Statistical analysis was performed using SPSS 26.0 for Windows (SPSS Inc., Chicago, IL, USA). A *P*-value < 0.05 was considered to be statistically significant.

### Propensity score matching (PSM)

**Fig. 3:**
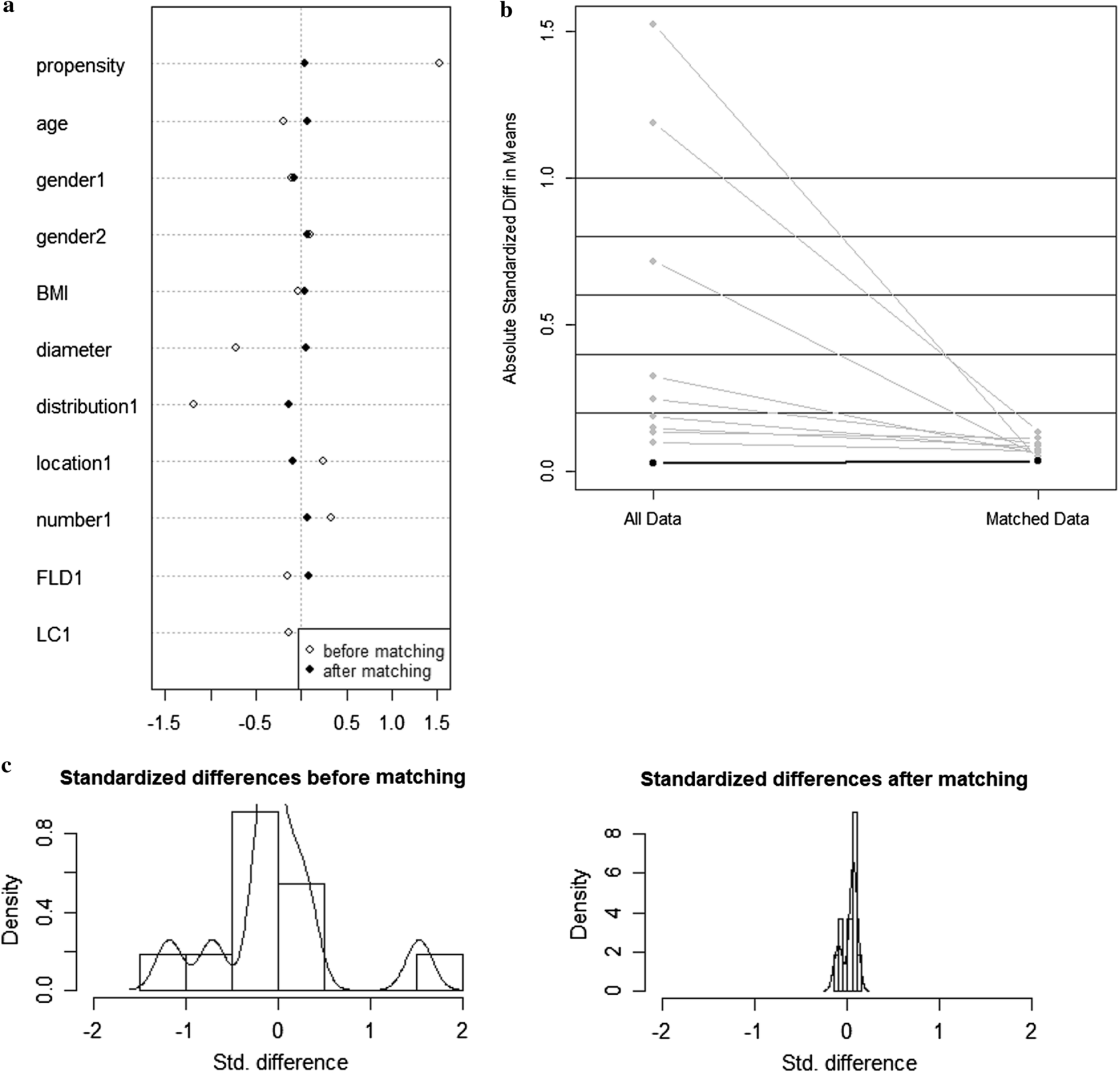
Plot of the propensity score-matched study. **a** Dot plot of standardized mean difference, **b** line plot of individual differences, **c** histogram of standardized mean differences (before and after)

In this study, PSM was used to adjust the baseline difference between the EN and LR groups. PSM analysis was conducted with SPSS 26.0 for Windows (SPSS 26, Inc., Chicago, IL, USA). The propensity score of each patient was analyzed by multivariate logistic regression. The width of 0.2 caliper was selected and the matching ratio was 1:1. The nearest neighbor method was used to match the groups (Fig. 3).

## Results

### Basic characteristics of patients

A total of 337 patients underwent surgery for HH during the study period. According to the exclusion criteria, 16 patients who underwent both LR and EN, 39 patients who underwent laparoscopic or robotic surgery, 5 patients with severe systemic diseases, 3 patients who received preoperative non-surgical treatment for HH and one patient who was converted from EN to LR were excluded. The remaining 273 patients were divided into the EN (n = 163) and LR (n = 110) groups. The indications for surgery in this study were: (i) tumor diameter ≥ 5 cm with obvious symptoms such as upper abdominal discomfort or pressure symptoms (n = 230); (ii) a rapid increase in a short term [annual growth ≥ 2 cm in diameter (n = 25)]; (iii) liver cancer could not be excluded (n = 18).

In the EN group, there were 124 women (76.1%) and 39 men (22.9%) with a mean age of 47.3 ± 9.0 years and a mean body mass index (BMI) of 23.3 ± 3.5 kg/m^2^. Twenty-four patients (14.7%) had fatty liver and three (1.8%) had liver cirrhosis. There were 64 patients with lesions near the major vessels (39.3%) and 142 patients had peripherally located lesions (87.1%). The mean diameter of the tumor was 9.4 ± 2.8 cm. A solitary tumor was observed in 97 patients (59.5%) and multiple tumors were observed in 66 patients (40.5%).

In the LR group, there were 79 women (71.8%) and 31 men (28.2%) with a mean age of 49.0 ± 10.2 years and a mean BMI of 23.4 ± 3.0 kg/m^2^. Twenty-two patients (20.0%) had fatty liver and four (3.6%) had liver cirrhosis. There were 30 patients with lesions close to the major blood vessels (27.3%) and 52 patients with peripherally located lesions (42.7%). The mean diameter of the tumor was 11.5 ± 4.2 cm. A single tumor was seen in 83 patients (75.5%) and multiple tumors were observed in 27 patients (24.5%).

**Fig. 4:**
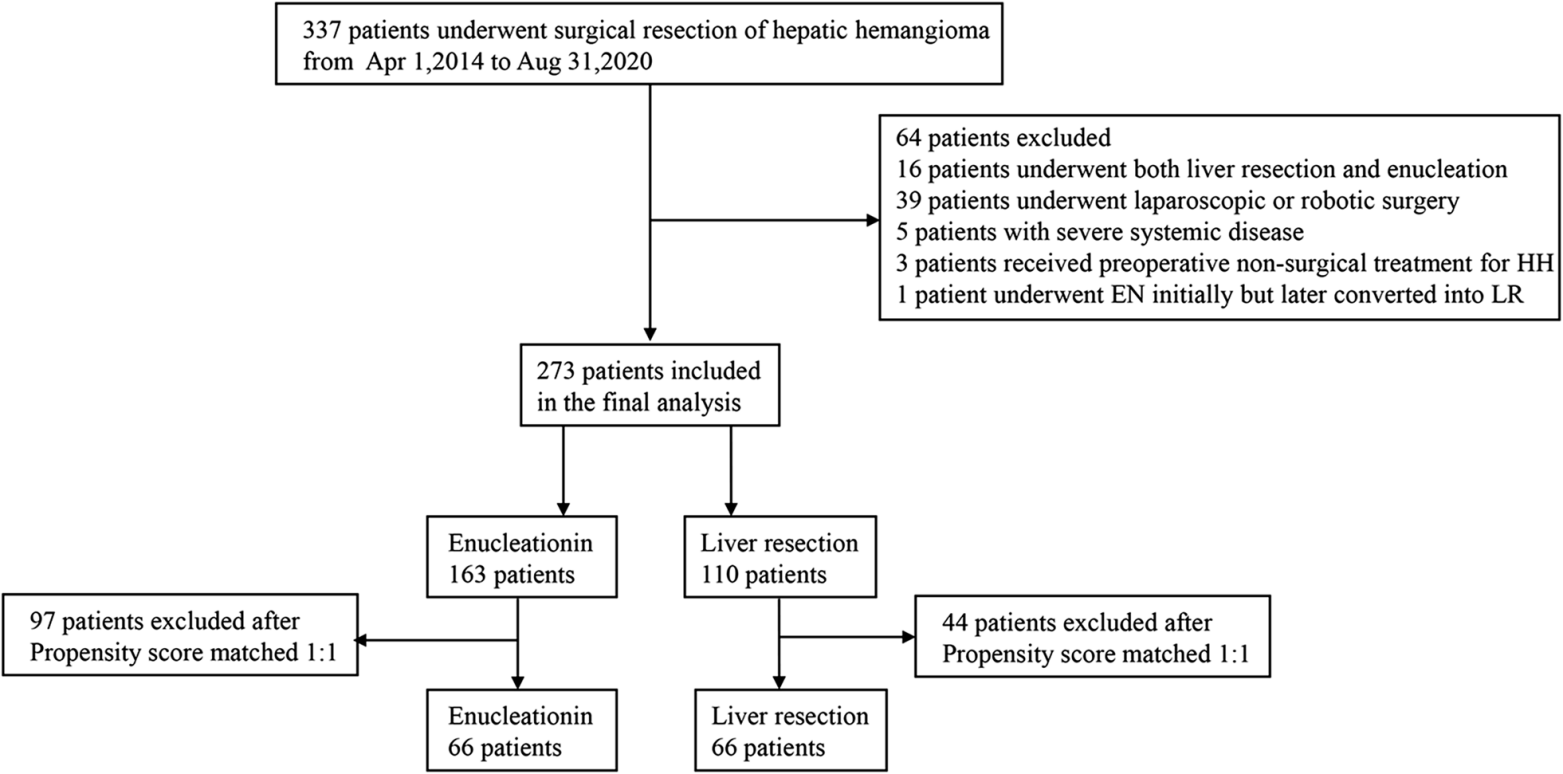
Flow diagram for the study on the efficacy of enucleation and liver resection

After PSM of age, sex, BMI, presence of liver cirrhosis or fatty liver, tumor location (proximal to massive vessels or not), tumor distribution (centrally located or peripherally located), tumor size and tumor number, 66 patients from each group were included (Table 1, Fig. 4).

**Table 1:** Demographics, comorbidities, and pre-operative data for patients undergoing surgery for hepatic hemangioma

Variable	Before propensity			After propensity		
	EN	LR	*P* value	EN	LR	*P* value
Number of patients (%)	163 (100)	110 (100)		66 (100)	66 (100)	
Age (years)	47.3 ± 9.0	49.0 ± 10.2	0.154	47.6 ± 9.4	47.0 ± 9.0	0.693
Gender						
Female	124 (76.1)	79 (71.8)	0.481	50 (75.8)	48 (72.7)	0.691
Male	39 (22.9)	31 (28.2)		16 (24.2)	18 (27.3)	
BMI (kg/m^2^)	23.3 ± 3.5	23.4 ± 3.0	0.817	23.4 ± 3.6	23.2 ± 2.9	0.827
Comorbidities						
Fatty liver	24 (14.7)	22 (20.0)	0.253	13 (19.7)	11 (16.7)	0.652
Liver cirrhosis	3 (1.8)	4 (3.6)	0.445	3 (4.5)	2 (3.0)	1.000
Preoperative data						
Tumor proximal to massive vessels			0.041*			0.561
Yes	64 (39.3)	30 (27.3)		17 (25.8)	20 (30.3)	
No	99 (60.7)	80 (72.7)		49 (74.2)	46 (69.7)	
Tumor distribution			< 0.001*			0.573
Centrally located	21 (12.9)	58 (42.7)		19 (28.8)	22 (33.3)	
Peripherally located	142 (87.1)	52 (57.3)		47 (71.2)	44 (66.7)	
Tumor size (cm)	9.4 ± 2.8	11.5 ± 4.2	< 0.001*	10.2 ± 2.9	10.1 ± 3.5	0.802
Tumor number			0.006^*^			
Single	97 (59.5)	83 (75.5)		42 (63.6)	44 (66.7)	0.715
Multiple	66 (40.5)	27 (24.5)		24 (36.4)	22 (33.3)	

Before propensity score matching, there were significant differences between the two groups in the location related to massive vessels, tumor distribution, radiographic tumor size, and tumor number between group E (n = 163) and group LR (n = 110). After matching, the four baseline variables were balanced (P > 0.05)

*LR* Liver resection, *EN* Enucleation, *BMI* Body mass index

*P < 0.05 was considered significant difference

### Independent factors affecting the choice of the surgical methods

As shown in Table 1, the four baseline variables, namely, tumor location, tumor distribution, tumor number, and tumor size were unbalanced before matching. To determine the factors affecting the choice of surgical procedure for HH, we included these variables in the univariate analysis. Previously, tumor size was transformed into binary variables based on the cut-off value that was revealed by the ROC curve (Youden index: 0.2468; associated criterion: ≤ 12.1 cm; sensitivity: 86.50%; specificity: 36.18%). The surgical method was taken as the dependent variable (LR = 0, EN = 1) which meant that EN was taken as the experimental group and LR as the control group. Other related factors (tumor location: not proximal to the major vessels = 0, proximal to the major vessels = 1; tumor distribution: peripherally located = 0, centrally located = 1; tumor size: ≤ 12.1 cm = 0, > 12.1 cm = 1; tumor number: single = 0, multiple = 1) were taken as independent variables or exposure factors. OR was equal to the ratio of exposure to non-exposure in EN group divided by the ratio of exposure to non-exposure in LR group. On univariate analysis with *α* = 0.05 as the test level, lesions proximal to the major vessels [OR: 1.724, 95% confidence interval (CI) 1.020–2.912] (lesions proximal to the major vessels were 1.724 times more likely to undergo EN than those far away from major vessels), centrally located lesions (OR: 0.133, 95% CI 0.073–0.240) (centrally located lesions were 0.133 times less likely to undergo EN than peripherally located lesions), tumors size > 12.1 cm (OR: 0.253, 95% CI 0.140–0.456) (tumors > 12.1 were 0.253 times less likely to undergo EN than tumors ≤ 12.1 cm) and multiple tumors (OR: 2.092, 95% CI 1.225–3.572) (multiple tumors were 2.092 times more likely to undergo EN than single tumors) were found to affect the choice of surgical method in univariate analysis and were included in multivariate logistic regression. The final results showed that centrally located lesions (OR: 0.131, 95% CI 0.070–0.244), tumors size > 12.1 cm (OR: 0.226, 95% CI 0.116–0.439) and multiple tumors (OR: 1.860, 95% CI 1.003–3.449) were independent factors affecting the choice of EN (Table 2). With these identified independent factors, the ROC was graphed independently for each variable and the combined variable (Fig. 2). The calibration of the model was evaluated by the Hosmer–Lemeshow good of fit test (*χ*^2^ = 12.174, *p* = 0.095).

**Table 2:** Univariate and multivariate Logistic regression analysis

Factors included	Univariate Logistic		Multivariate logistic	
	O^^^R	95% CI	O^^^R	95% CI
Proximal to the major vessels	1.724	1.020–2.912	1.599	0.869–2.940
Centrally located lesions	0.133	0.073–0.240	0.131	0.070–0.244
Tumors size > 12.1 cm	0.253	0.140–0.456	0.226	0.116–0.439
Multiple tumors	2.092	1.225–3.572	1.860	1.003–3.449

EN was taken as the experimental group and LR as the control group. Proximal to the major vessels, centrally located, tumor size > 12.1 cm and multiple tumors were taken as independent variables or exposure factors. OR was equal to the ratio of exposure to non-exposure in EN group divided by the ratio of exposure to non-exposure in LR group

### Comparison of the perioperative outcomes of two groups after PSM

There was no significant difference in median operative time (156 min vs. 195 min), median estimated blood loss (200 ml vs. 220 ml), or the blood transfusion rate (33.3% vs. 33.3%) between the EN and LR groups. There was no significant difference in the mean postoperative feeding (3.1 d vs. 3.3 d), mean abdominal drain duration (5.9 d vs. 6.0 d), or mean postoperative hospital stay (9.5 d vs. 9.0 d) between the two groups. Postoperative complications including ascites (6.1% vs. 4.5%), abdominal infection (6.1% vs. 4.5%), biliary fistula (1.5% vs. 3.0%) and postoperative bleeding (1.5 vs. 4.5%) were comparable between the two groups (Table 3). There were no perioperative deaths.

**Table 3:** Peri-operative data for patients undergoing surgical resection of hepatic hemangioma in this cohort

Variable	Before propensity			After propensity		
	EN	LR	*P* value	EN	LR	*P* value
Number of patients (%)	163 (100)	110 (100)		66 (100)	66 (100)	
Operation time (minutes)	150 (130–200)	195 (149–246)	< 0.001*	156 (130–210)	195 (139–238)	0.156
Estimated blood loss (ml)	200 (100–300)	300 (100–450)	0.048*	200 (150–425)	220 (100–360)	0.423
Blood transfusion rate	37 (22.7)	38 (34.5)	0.031*	22 (33.3)	22 (33.3)	1.000
Postoperative data						
Postoperative feeding (days)	3.2 ± 1.2	3.5 ± 1.3	0.027*	3.1 ± 1.0	3.3 ± 1.0	0.460
Abdominal drains (days)	5.8 ± 1.9	6.2 ± 2.6	0.036*	5.9 ± 1.9	6.0 ± 1.9	0.643
Postoperative hospital stay (days)	9.1 ± 2.5	9.2 ± 3.3	0.700	9.5 ± 2.6	9.0 ± 1.9	0.206
Complication						
Ascites	7 (4.3)	5 (4.5)	1.000	4 (6.1)	3 (4.5)	1.000
Abdominal infection	10 (6.1)	10 (9.1)	0.358	4 (6.1)	3 (4.5)	1.000
Bile fistula	1 (0.6)	5 (4.5)	0.041*	1 (1.5)	2 (3.0)	1.000
Bleeding	2 (1.1)	4 (2.8)	0.224	1 (1.5)	3 (4.5)	0.619

*LR* liver resection, *EN* enucleation

*P < 0.05 was considered significant difference

## Discussion

Based on the univariate and multivariate analysis, we found that tumor size > 12.1 cm, centrally located lesions, and multiple tumors were independent factors affecting the choice of EN. After PSM, there were no significant differences in the efficacy of EN or LR.

In the pre-operative data, we found that tumor size in EN group was significantly smaller than that in LR group which suggests that LR was preferred for giant hemangiomas, especially for HH sized > 12.1 cm. Giant HH occupies more than one liver segment, so the cut surface is wide and the risk of bleeding is high after enucleation. Moreover, once the capsule is injured during EN, the bleeding is difficult to control. On the contrary, during anatomical LR, the blood vessels supplying the liver segment or lobe are ligated first, which ensures the surgical safety. Liu et al. proposed in a retrospective study that large hemangiomas, especially those extremely giant liver hemangioma > 20 cm were more suitable for LR because of less use of Pringle maneuver [19]. At the same time, due to the complete preservation of the vessels of the remaining liver, the function of the remnant liver is preserved. Borgonovo et al. [17] deemed that to achieve lower morbidity and blood loss, in the case of large HHs, typical liver resection was the best choice and EN was an option when a lesion was small. However, it is undeniable that anatomical LR requires higher surgical skills, so only experienced surgeons who can deal with complex blood vessels and anatomical variations should undertake LR.

EN may be preferred for lesions located peripherally where the intraoperative exposure was relatively sufficient to allow EN. The centrally distributed HHs embedded in the liver parenchyma are difficult to remove with EN. For example, HH occupying S7 and S8 was very deep and close to the top of the diaphragm, so the patient received anatomical right hemihepatectomy and was discharged successfully without any complications (Fig. 1). Orhan et al. considered that if such tumor location as mentioned above precludes safe EN, anatomic resection is preferred [22].

In this study, patients with multiple HH were more inclined to receive EN. This was because the multiple lesions were widely distributed and LR would have resulted in more loss of liver parenchyma, and a higher incidence of postoperative liver failure and other complications [22]. But if the multiple tumors were located in the same or adjoining liver segments, it was easier to perform LR along anatomical planes rather than performing EN for each lesion. In some cases, if multiple HHs are widely distributed (distributed in different hepatic lobes and segments), then EN may be combined with LR, or a multi-stage operation can be performed if the future liver remnant (FLR) is insufficient.

Since the first use of EN in 1988, many studies found EN to have better outcomes for HH [12–15, 23]. There are fewer blood vessels along with the capsule so the vascular and bile duct injury is avoided to the greatest extent [24, 25]. Also, the normal liver tissue is preserved as much as possible, there by reducing the risk of postoperative liver dysfunction [19]. In this study, EN was indeed superior to LR, in terms of peri-operative data such as operative time, estimated blood loss, postoperative feeding, abdominal drains, and postoperative hospital stay before PSM. However, after PSM the outcomes of EN and LR groups were similar. Zhang et al. [26] in their study of 86 cases of HH with a diameter > 10 cm found that there was no significant difference in operative time, hepatic inflow occlusion, blood loss or complications between the two groups. It's worth noting that these previous studies had some limitations: the sample size was small and there was no separate analysis of the impact of the size, location and number of HHs on the choice of surgical methods. In this study, we believe that it was unreasonable to statistically analyze the non-comparable data of two groups. So we applied PSM, a method that could eliminate the confounding bias and make the study more scientific. Each procedure has its own advantages and disadvantages. As long as we reasonably master the technique of EN and LR instead of blindly pursuing a certain surgical method, we could use both these procedures depending the tumor size, location and number in order to the best outcomes.

There are some limitations to this study. First, this is a retrospective single-center study instead of an intention-to-treat (ITT) analysis and the choice of surgical procedure may be influenced by the experience of surgeons. Second, the number of patients in each group after PSM was relatively small. Hence, future prospective studies with larger sample sizes and long-term follow-up are needed to further validate our results.

## Conclusions

Patients with symptomatic giant HHs having peripherally located lesions, tumors size less than or equal to 12.1 cm and multiple tumors were more inclined to receive EN by open surgery. After PSM analysis, there was no enough evidence to prove that there were significant differences in the efficacy of EN or LR. To give full play to the superiority of the two surgical methods, above-mentioned factors must be taken into account.

## Data Availability

The datasets used and analyzed during the current study are available from the corresponding author on reasonable request.

## Abbreviations

HH: Hepatic hemangioma
LR: Liver resection
EN: Enucleation
PSM: Propensity score matching
CT: Computed tomography
MRI: Magnetic resonance imaging
FLR: Future liver remnant
ICGR15: Indocyanine green 15-min retention test
BMI: Body mass index
AUC: Area under the curve
ROC: Receiver operating characteristic
OR: Odds ratio
FLD: Fatty liver disease
LC: Liver cirrhosis
